# 2-[2,6-Bis(propan-2-yl)phen­yl]-1,3-di­cyclo­hexyl­guanidine

**DOI:** 10.1107/S1600536814011611

**Published:** 2014-06-18

**Authors:** Tomáš Chlupatý, Zdeňka Padělková

**Affiliations:** a Department of General and Inorganic Chemistry, Faculty of Chemical Technology, University of Pardubice, Studentská 573, 53210 Pardubice, Czech Republic

## Abstract

In the title asymmetric di­cyclo­hexyl­phenyl­guanidine, C_25_H_41_N_3_, the central guanidine C atom deviates by only 0.004 (2) Å from the central plane defined by the three N atoms. The benzene and the cyclo­hexyl rings are rotated out of the central plane of the N_3_C unit by 85.63 (12)° (benzene) and 51.52 (9) and 49.37 (12)° (cyclohexyl). The crystal packing features only by van der Waals inter­actions.

## Related literature   

For similar structures of various related compounds, see: Shen *et al.* (2011 ▶); Ghosh *et al.* (2008 ▶); Yıldırım *et al.* (2007 ▶); Brazeau *et al.* (2012 ▶); Han & Huynh (2009 ▶); Tanatani *et al.* (1998 ▶); Zhang *et al.* (2009 ▶); Boere *et al.* (2000 ▶). For standard bond lengths, see: Allen *et al.* (1987 ▶).
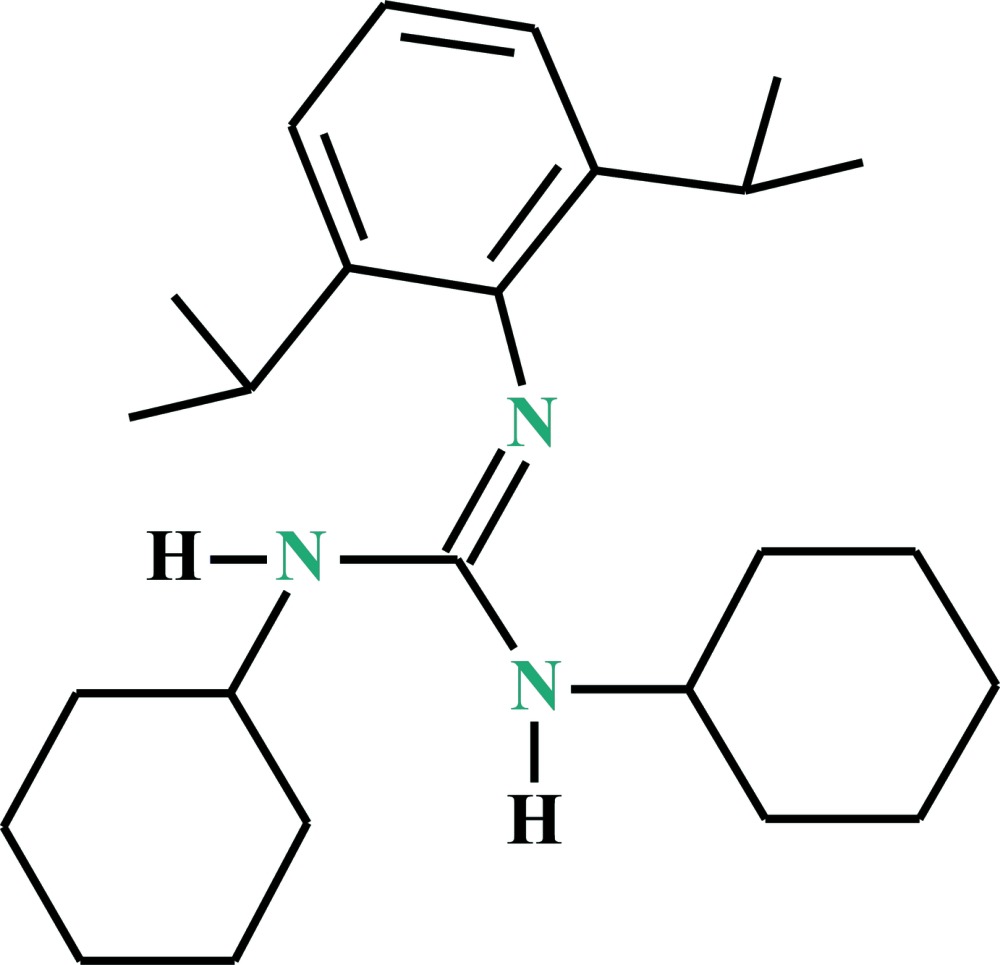


## Experimental   

### 

#### Crystal data  

C_25_H_41_N_3_
*M* *_r_* = 383.61
Monoclinic, 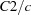

*a* = 30.9001 (3) Å
*b* = 9.9442 (5) Å
*c* = 18.5260 (3) Å
β = 124.962 (3)°
*V* = 4665.3 (3) Å^3^
*Z* = 8
Mo *K*α radiation
μ = 0.06 mm^−1^
*T* = 150 K
0.45 × 0.18 × 0.18 mm

#### Data collection  

Bruker–Nonius KappaCCD area-detector diffractometer
Absorption correction: gaussian (Coppens, 1970 ▶) *T*_min_ = 0.982, *T*_max_ = 0.991
40512 measured reflections
5336 independent reflections
3272 reflections with *I* > 2σ(*I*)
*R*_int_ = 0.098

#### Refinement  

*R*[*F* ^2^ > 2σ(*F* ^2^)] = 0.060
*wR*(*F* ^2^) = 0.137
*S* = 1.06
5336 reflections
253 parameters
H-atom parameters constrained
Δρ_max_ = 0.41 e Å^−3^
Δρ_min_ = −0.37 e Å^−3^

### 

Data collection: *COLLECT* (Hooft, 1998 ▶) and *DENZO* (Otwinowski & Minor, 1997 ▶); cell refinement: *COLLECT* and *DENZO*; data reduction: *COLLECT* and *DENZO*; program(s) used to solve structure: *SIR92* (Altomare *et al.*, 1994 ▶); program(s) used to refine structure: *SHELXL97* (Sheldrick, 2008 ▶); molecular graphics: *PLATON* (Spek, 2009 ▶); software used to prepare material for publication: *SHELXL97*.

## Supplementary Material

Crystal structure: contains datablock(s) I. DOI: 10.1107/S1600536814011611/kp2469sup1.cif

Structure factors: contains datablock(s) I. DOI: 10.1107/S1600536814011611/kp2469Isup2.hkl

Supporting information file. DOI: 10.1107/S1600536814011611/kp2469Isup3.cml

CCDC reference: 1004128

Additional supporting information:  crystallographic information; 3D view; checkCIF report

## Figures and Tables

**Fig. 1.**
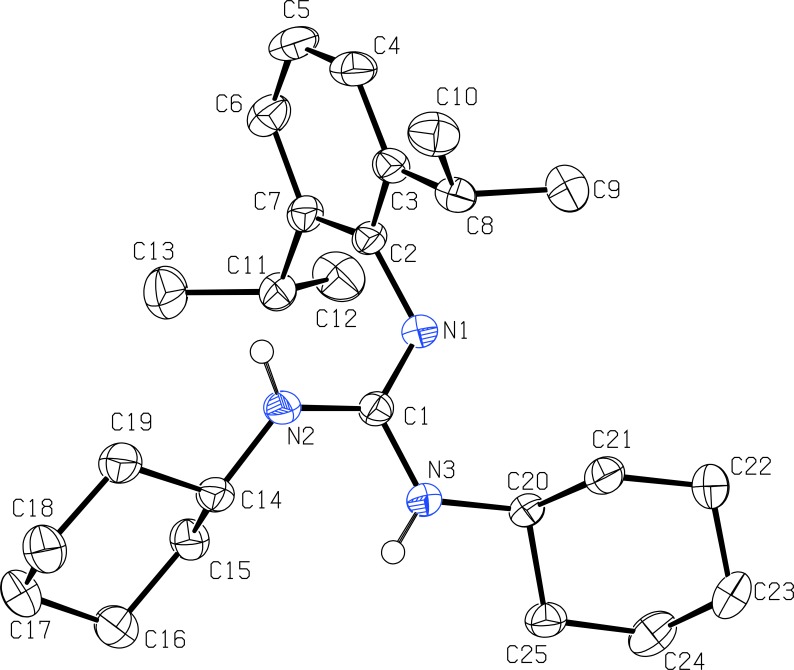
View of the title compound with the displacement ellipsoids shown at the 50% probability level. The H atoms are shown with arbitrary radii.

## supplementary crystallographic information

### S1. Comment

The determination of the structure of title compound (Fig. 1) was carried out in order to compare the essential structural parametes of this type of guanidine with other structures which will be isolated from its reactivity investigation e.g. both protonation and deprotonation reactions leading presumably to guanidinium, guanidinate(-) or guanidinate(2-) salts. The guanidinium salts and guanidinates are common species in nowadays chemistry and can be used as versatile ligands. Guanidine can be used as a precursor of the desired products by reactions with an acid or a base. Asymmetric guanidinates or guanidinium salts which are frequently tested for mentioned applications contain usually one or more phenyl rings facilitating crystallization of products. Except of three examples of phenyl substituted benzimidazol amines (Shen *et al.* (2011); Ghosh *et al.* (2008); Yıldırım *et al.* (2007)), there are five examples of acyclic phenyl substituted guanidines (see below). In this series the title compound, bis(cyclohexyl-2,6-(diisopropyl)phenyl (Dipp) substituted guanidine, is together with *N''*-methyl-*N,N'*-diphenylguanidine (Tanatani *et al.* (1998)) and 1-cyclohexyl-2,3-diphenylguanidine (Zhang *et al.* (2009)) the only representative of asymmetric species reported so far. The delocalization of π-electrons and thus the presence of so-called Y-aromaticity described for protonated or deprotonated guanidines is not taking part in these compounds. The degree of multiple C–N bonds localization is strongly dependent to the steric as well as electronic feature of all three substituents of the fundamental N–C(N)–N skeleton. The C=N double bond in I is localized on the connection of the central skeleton with the Dipp substituent with interatomic distance of 1.289 (2) Å and the rest of C–N bonds from the centre of the structure can be attributed to regular C–N single bonds ((Allen *et al.* (1987)). The same structural arrangements were found by Brazeau *et al.* (2012) for 1-(2,6-diisopropylphenyl)-2,3-dimesitylguanidine, Han *et al.* (2009) for *N,N',N''*-tris(2,6-dimethylphenyl)guanidine, Tanatani *et al.* (1998) for *N''*-methyl-*N,N'*-diphenylguanidine and Zhang *et al.* (2009) for 1-cyclohexyl-2,3-diphenylguanidine. On the contrary, the central motif of highly stericaly crowded (Boere *et al.* (2000)), *N,N',N''*-tris(2,6-di-isopropylphenyl)guanidine reveals much lower π-electron delocalization than I and other reported species due to steric demands of Dipp substituents. The central N_3_C skeleton is approaching the ideally planar arrangement similarly as in the cases of the rest of phenylguanidinates mentioned above. The N–C–N angles in all compounds are close to 120° with the small deviation of the interatomic angles of NH-C-NH fragment - in the case of I the angle N2–C1–N3 being about 4° sharper. There are no close contacts within the monoclinic *C*2/*c* unit cell of I.

### S2. Refinement

All the hydrogens were discernible in the difference electron density map. However, all the hydrogens were situated into idealized positions and refined riding on their parent C or N atoms, with N–H = 0.86 Å, C–H = 0.93 Å for aromatic H atoms, with U(H) = 1.2U*_eq_*(C/N) for the NH group and U(H) = 1.5U*_eq_*(C/N) for other H atoms, respectively.

### Crystal data

**Table:**

C_25_H_41_N_3_	*F*(000) = 1696
*M**_r_* = 383.61	*D*_x_ = 1.092 Mg m^−^^3^
Monoclinic, *C*2/*c*	Mo *K*α radiation, λ = 0.71073 Å
Hall symbol: -C 2yc	Cell parameters from 40662 reflections
*a* = 30.9001 (3) Å	θ = 1–27.5°
*b* = 9.9442 (5) Å	µ = 0.06 mm^−^^1^
*c* = 18.5260 (3) Å	*T* = 150 K
β = 124.962 (3)°	Needle, colourless
*V* = 4665.3 (3) Å^3^	0.45 × 0.18 × 0.18 mm
*Z* = 8	

### Data collection

**Table:**

Bruker–Nonius KappaCCD area-detector diffractometer	5336 independent reflections
Radiation source: fine-focus sealed tube	3272 reflections with *I* > 2σ(*I*)
Graphite monochromator	*R*_int_ = 0.098
Detector resolution: 9.091 pixels mm^-1^	θ_max_ = 27.5°, θ_min_ = 2.2°
φ and ω scans to fill the Ewald sphere	*h* = −40→37
Absorption correction: gaussian (Coppens, 1970)	*k* = −12→12
*T*_min_ = 0.982, *T*_max_ = 0.991	*l* = −24→24
40512 measured reflections	

### Refinement

**Table:**

Refinement on *F*^2^	Primary atom site location: structure-invariant direct methods
Least-squares matrix: full	Secondary atom site location: difference Fourier map
*R*[*F*^2^ > 2σ(*F*^2^)] = 0.060	Hydrogen site location: inferred from neighbouring sites
*wR*(*F*^2^) = 0.137	H-atom parameters constrained
*S* = 1.06	*w* = 1/[σ^2^(*F*_o_^2^) + (0.0373*P*)^2^ + 6.0132*P*] where *P* = (*F*_o_^2^ + 2*F*_c_^2^)/3
5336 reflections	(Δ/σ)_max_ < 0.001
253 parameters	Δρ_max_ = 0.41 e Å^−^^3^
0 restraints	Δρ_min_ = −0.37 e Å^−^^3^

### Special details

**Table:**

Geometry. All esds (except the esd in the dihedral angle between two l.s. planes) are estimated using the full covariance matrix. The cell esds are taken into account individually in the estimation of esds in distances, angles and torsion angles; correlations between esds in cell parameters are only used when they are defined by crystal symmetry. An approximate (isotropic) treatment of cell esds is used for estimating esds involving l.s. planes.
Refinement. Refinement of F^2^ against ALL reflections. The weighted R-factor wR and goodness of fit S are based on F^2^, conventional R-factors R are based on F, with F set to zero for negative F^2^. The threshold expression of F^2^ > 2sigma(F^2^) is used only for calculating R-factors(gt) etc. and is not relevant to the choice of reflections for refinement. R-factors based on F^2^ are statistically about twice as large as those based on F, and R- factors based on ALL data will be even larger.

### Fractional atomic coordinates and isotropic or equivalent isotropic displacement parameters (Å^2^)

**Table:**

	*x*	*y*	*z*	*U*_iso_*/*U*_eq_	
N1	0.85963 (6)	−0.00660 (16)	0.09390 (11)	0.0249 (4)	
N2	0.91824 (6)	0.12199 (17)	0.22129 (11)	0.0272 (4)	
H2	0.9424	0.1231	0.2117	0.033*	
C1	0.87631 (7)	0.03532 (18)	0.17160 (13)	0.0230 (4)	
N3	0.85204 (6)	−0.00799 (17)	0.21031 (11)	0.0281 (4)	
H3	0.8618	0.0247	0.2605	0.034*	
C7	0.86512 (7)	0.1607 (2)	0.00225 (13)	0.0259 (4)	
C2	0.88288 (7)	0.0413 (2)	0.05185 (13)	0.0242 (4)	
C3	0.92155 (7)	−0.0387 (2)	0.05466 (13)	0.0268 (4)	
C4	0.93881 (8)	−0.0019 (2)	0.00274 (14)	0.0335 (5)	
H4	0.9635	−0.0554	0.0027	0.040*	
C20	0.81023 (8)	−0.10825 (19)	0.16858 (13)	0.0246 (4)	
H20	0.7872	−0.0884	0.1054	0.029*	
C25	0.77745 (8)	−0.0977 (2)	0.20611 (14)	0.0292 (5)	
H25A	0.8000	−0.1130	0.2692	0.035*	
H25B	0.7628	−0.0079	0.1960	0.035*	
C8	0.94246 (8)	−0.1630 (2)	0.11264 (14)	0.0305 (5)	
H8	0.9429	−0.1434	0.1649	0.037*	
C19	0.97612 (8)	0.2886 (2)	0.32971 (14)	0.0347 (5)	
H19A	0.9757	0.3364	0.2837	0.042*	
H19B	1.0052	0.2252	0.3564	0.042*	
C15	0.87852 (8)	0.3080 (2)	0.25502 (14)	0.0309 (5)	
H15A	0.8737	0.3595	0.2064	0.037*	
H15B	0.8465	0.2567	0.2329	0.037*	
C11	0.82506 (8)	0.2511 (2)	0.00116 (14)	0.0284 (5)	
H11	0.8238	0.2248	0.0509	0.034*	
C14	0.92473 (7)	0.2116 (2)	0.28959 (13)	0.0266 (4)	
H14	0.9274	0.1562	0.3357	0.032*	
C21	0.83129 (9)	−0.2505 (2)	0.17984 (16)	0.0347 (5)	
H21A	0.8517	−0.2565	0.1551	0.042*	
H21B	0.8545	−0.2718	0.2421	0.042*	
C10	0.99868 (9)	−0.2004 (2)	0.14496 (16)	0.0408 (6)	
H10A	0.9990	−0.2301	0.0960	0.049*	
H10B	1.0112	−0.2714	0.1876	0.049*	
H10C	1.0212	−0.1233	0.1716	0.049*	
C6	0.88416 (8)	0.1932 (2)	−0.04814 (14)	0.0337 (5)	
H6	0.8726	0.2715	−0.0817	0.040*	
C5	0.91994 (8)	0.1121 (2)	−0.04892 (14)	0.0368 (5)	
H5	0.9314	0.1341	−0.0842	0.044*	
C16	0.88758 (9)	0.4038 (2)	0.32643 (15)	0.0374 (5)	
H16A	0.8582	0.4660	0.3017	0.045*	
H16B	0.8893	0.3532	0.3728	0.045*	
C24	0.73288 (8)	−0.2005 (2)	0.16355 (16)	0.0394 (6)	
H24A	0.7143	−0.1963	0.1915	0.047*	
H24B	0.7080	−0.1777	0.1019	0.047*	
C12	0.77015 (9)	0.2305 (3)	−0.08243 (16)	0.0453 (6)	
H12A	0.7456	0.2881	−0.0812	0.054*	
H12B	0.7597	0.1384	−0.0861	0.054*	
H12C	0.7703	0.2521	−0.1328	0.054*	
C23	0.75267 (10)	−0.3431 (2)	0.17037 (17)	0.0425 (6)	
H23A	0.7228	−0.4035	0.1373	0.051*	
H23B	0.7734	−0.3713	0.2315	0.051*	
C9	0.90576 (10)	−0.2825 (2)	0.06648 (16)	0.0441 (6)	
H9A	0.9059	−0.3079	0.0166	0.053*	
H9B	0.8706	−0.2579	0.0470	0.053*	
H9C	0.9175	−0.3569	0.1066	0.053*	
C13	0.84053 (10)	0.3987 (2)	0.01292 (19)	0.0496 (7)	
H13A	0.8382	0.4305	−0.0381	0.059*	
H13B	0.8761	0.4088	0.0640	0.059*	
H13C	0.8171	0.4499	0.0206	0.059*	
C17	0.93828 (9)	0.4821 (2)	0.36433 (16)	0.0429 (6)	
H17A	0.9351	0.5393	0.3191	0.052*	
H17B	0.9442	0.5394	0.4116	0.052*	
C18	0.98491 (9)	0.3882 (3)	0.39960 (15)	0.0427 (6)	
H18A	0.9908	0.3394	0.4499	0.051*	
H18B	1.0163	0.4410	0.4196	0.051*	
C22	0.78632 (10)	−0.3517 (2)	0.13444 (18)	0.0435 (6)	
H22A	0.8005	−0.4419	0.1432	0.052*	
H22B	0.7645	−0.3338	0.0717	0.052*	

### Atomic displacement parameters (Å^2^)

**Table:**

	*U*^11^	*U*^22^	*U*^33^	*U*^12^	*U*^13^	*U*^23^
N1	0.0297 (9)	0.0247 (9)	0.0258 (9)	−0.0024 (7)	0.0191 (8)	−0.0014 (7)
N2	0.0262 (9)	0.0324 (9)	0.0287 (9)	−0.0067 (8)	0.0190 (8)	−0.0076 (8)
C1	0.0243 (10)	0.0210 (10)	0.0273 (11)	0.0013 (8)	0.0169 (9)	0.0007 (8)
N3	0.0362 (9)	0.0287 (9)	0.0271 (9)	−0.0093 (8)	0.0225 (8)	−0.0058 (8)
C7	0.0235 (10)	0.0299 (11)	0.0242 (10)	−0.0055 (9)	0.0136 (9)	−0.0031 (9)
C2	0.0236 (10)	0.0288 (10)	0.0224 (10)	−0.0069 (8)	0.0145 (8)	−0.0039 (8)
C3	0.0268 (10)	0.0314 (11)	0.0235 (11)	−0.0056 (9)	0.0152 (9)	−0.0063 (9)
C4	0.0311 (11)	0.0449 (13)	0.0316 (12)	−0.0006 (10)	0.0222 (10)	−0.0034 (10)
C20	0.0301 (10)	0.0234 (10)	0.0252 (10)	−0.0020 (9)	0.0188 (9)	0.0010 (8)
C25	0.0328 (11)	0.0293 (11)	0.0335 (12)	0.0028 (9)	0.0238 (10)	0.0015 (9)
C8	0.0369 (12)	0.0305 (11)	0.0309 (12)	0.0002 (9)	0.0234 (10)	−0.0032 (9)
C19	0.0282 (11)	0.0446 (13)	0.0317 (12)	−0.0083 (10)	0.0175 (10)	−0.0073 (10)
C15	0.0283 (11)	0.0333 (12)	0.0286 (12)	−0.0046 (9)	0.0148 (9)	−0.0051 (9)
C11	0.0334 (11)	0.0262 (10)	0.0280 (11)	−0.0024 (9)	0.0190 (10)	0.0007 (9)
C14	0.0281 (11)	0.0310 (11)	0.0225 (10)	−0.0043 (9)	0.0155 (9)	−0.0029 (9)
C21	0.0452 (13)	0.0269 (11)	0.0485 (14)	−0.0009 (10)	0.0366 (12)	−0.0002 (10)
C10	0.0408 (13)	0.0436 (14)	0.0407 (14)	0.0086 (11)	0.0248 (11)	0.0031 (11)
C6	0.0318 (11)	0.0399 (12)	0.0285 (12)	−0.0044 (10)	0.0168 (10)	0.0046 (10)
C5	0.0343 (12)	0.0545 (14)	0.0312 (12)	−0.0054 (11)	0.0244 (10)	0.0010 (11)
C16	0.0398 (12)	0.0374 (12)	0.0375 (13)	−0.0019 (10)	0.0237 (11)	−0.0060 (10)
C24	0.0296 (11)	0.0474 (14)	0.0449 (14)	−0.0018 (11)	0.0236 (11)	0.0057 (11)
C12	0.0345 (13)	0.0515 (15)	0.0456 (15)	0.0038 (11)	0.0205 (12)	−0.0053 (12)
C23	0.0496 (14)	0.0355 (12)	0.0530 (15)	−0.0130 (11)	0.0357 (13)	−0.0007 (11)
C9	0.0550 (15)	0.0369 (13)	0.0448 (15)	−0.0077 (12)	0.0313 (13)	−0.0082 (11)
C13	0.0585 (16)	0.0332 (13)	0.0658 (18)	−0.0030 (12)	0.0408 (15)	−0.0041 (12)
C17	0.0500 (14)	0.0388 (13)	0.0402 (14)	−0.0123 (12)	0.0260 (12)	−0.0155 (11)
C18	0.0353 (12)	0.0523 (15)	0.0336 (13)	−0.0167 (11)	0.0156 (11)	−0.0158 (11)
C22	0.0659 (16)	0.0254 (11)	0.0565 (16)	−0.0099 (11)	0.0451 (14)	−0.0074 (11)

### Geometric parameters (Å, º)

**Table:**

N1—C1	1.289 (2)	C14—H14	0.9798
N1—C2	1.411 (2)	C21—C22	1.521 (3)
N2—C1	1.379 (2)	C21—H21A	0.9702
N2—C14	1.465 (2)	C21—H21B	0.9699
N2—H2	0.8602	C10—H10A	0.9599
C1—N3	1.370 (2)	C10—H10B	0.9600
N3—C20	1.455 (2)	C10—H10C	0.9601
N3—H3	0.8599	C6—C5	1.375 (3)
C7—C6	1.397 (3)	C6—H6	0.9299
C7—C2	1.406 (3)	C5—H5	0.9301
C7—C11	1.521 (3)	C16—C17	1.515 (3)
C2—C3	1.412 (3)	C16—H16A	0.9700
C3—C4	1.390 (3)	C16—H16B	0.9701
C3—C8	1.518 (3)	C24—C23	1.520 (3)
C4—C5	1.380 (3)	C24—H24A	0.9698
C4—H4	0.9299	C24—H24B	0.9699
C20—C21	1.521 (3)	C12—H12A	0.9598
C20—C25	1.526 (3)	C12—H12B	0.9601
C20—H20	0.9799	C12—H12C	0.9601
C25—C24	1.523 (3)	C23—C22	1.525 (3)
C25—H25A	0.9700	C23—H23A	0.9701
C25—H25B	0.9701	C23—H23B	0.9700
C8—C9	1.522 (3)	C9—H9A	0.9597
C8—C10	1.524 (3)	C9—H9B	0.9602
C8—H8	0.9798	C9—H9C	0.9601
C19—C14	1.519 (3)	C13—H13A	0.9600
C19—C18	1.526 (3)	C13—H13B	0.9601
C19—H19A	0.9700	C13—H13C	0.9600
C19—H19B	0.9699	C17—C18	1.515 (3)
C15—C16	1.521 (3)	C17—H17A	0.9699
C15—C14	1.523 (3)	C17—H17B	0.9701
C15—H15A	0.9700	C18—H18A	0.9700
C15—H15B	0.9700	C18—H18B	0.9702
C11—C12	1.519 (3)	C22—H22A	0.9700
C11—C13	1.520 (3)	C22—H22B	0.9700
C11—H11	0.9801		
			
C1—N1—C2	120.19 (16)	C20—C21—H21B	109.4
C1—N2—C14	124.63 (16)	H21A—C21—H21B	108.1
C1—N2—H2	117.7	C8—C10—H10A	109.3
C14—N2—H2	117.7	C8—C10—H10B	109.5
N1—C1—N3	119.62 (17)	H10A—C10—H10B	109.5
N1—C1—N2	124.68 (17)	C8—C10—H10C	109.6
N3—C1—N2	115.69 (17)	H10A—C10—H10C	109.5
C1—N3—C20	121.52 (16)	H10B—C10—H10C	109.5
C1—N3—H3	119.3	C5—C6—C7	121.4 (2)
C20—N3—H3	119.2	C5—C6—H6	119.2
C6—C7—C2	118.41 (18)	C7—C6—H6	119.4
C6—C7—C11	120.38 (19)	C6—C5—C4	119.83 (19)
C2—C7—C11	121.19 (17)	C6—C5—H5	120.1
C7—C2—N1	120.96 (17)	C4—C5—H5	120.0
C7—C2—C3	120.35 (17)	C17—C16—C15	110.58 (18)
N1—C2—C3	118.49 (17)	C17—C16—H16A	109.3
C4—C3—C2	118.59 (19)	C15—C16—H16A	109.4
C4—C3—C8	121.83 (18)	C17—C16—H16B	109.8
C2—C3—C8	119.57 (17)	C15—C16—H16B	109.6
C5—C4—C3	121.3 (2)	H16A—C16—H16B	108.1
C5—C4—H4	119.3	C23—C24—C25	112.42 (18)
C3—C4—H4	119.4	C23—C24—H24A	109.2
N3—C20—C21	112.63 (16)	C25—C24—H24A	109.3
N3—C20—C25	109.28 (16)	C23—C24—H24B	109.0
C21—C20—C25	110.17 (16)	C25—C24—H24B	108.9
N3—C20—H20	108.2	H24A—C24—H24B	107.9
C21—C20—H20	108.2	C11—C12—H12A	109.3
C25—C20—H20	108.2	C11—C12—H12B	109.5
C24—C25—C20	110.97 (17)	H12A—C12—H12B	109.5
C24—C25—H25A	109.3	C11—C12—H12C	109.6
C20—C25—H25A	109.4	H12A—C12—H12C	109.5
C24—C25—H25B	109.5	H12B—C12—H12C	109.5
C20—C25—H25B	109.5	C24—C23—C22	111.05 (18)
H25A—C25—H25B	108.1	C24—C23—H23A	109.5
C3—C8—C9	111.11 (18)	C22—C23—H23A	109.2
C3—C8—C10	113.82 (17)	C24—C23—H23B	109.4
C9—C8—C10	110.19 (18)	C22—C23—H23B	109.6
C3—C8—H8	107.2	H23A—C23—H23B	108.1
C9—C8—H8	107.1	C8—C9—H9A	109.5
C10—C8—H8	107.1	C8—C9—H9B	109.3
C14—C19—C18	111.73 (17)	H9A—C9—H9B	109.5
C14—C19—H19A	109.5	C8—C9—H9C	109.6
C18—C19—H19A	109.5	H9A—C9—H9C	109.5
C14—C19—H19B	109.0	H9B—C9—H9C	109.4
C18—C19—H19B	109.1	C11—C13—H13A	109.7
H19A—C19—H19B	107.9	C11—C13—H13B	109.3
C16—C15—C14	111.56 (17)	H13A—C13—H13B	109.5
C16—C15—H15A	109.3	C11—C13—H13C	109.4
C14—C15—H15A	109.3	H13A—C13—H13C	109.5
C16—C15—H15B	109.4	H13B—C13—H13C	109.5
C14—C15—H15B	109.3	C16—C17—C18	111.0 (2)
H15A—C15—H15B	107.9	C16—C17—H17A	109.6
C12—C11—C13	110.6 (2)	C18—C17—H17A	109.5
C12—C11—C7	111.03 (17)	C16—C17—H17B	109.3
C13—C11—C7	112.47 (17)	C18—C17—H17B	109.4
C12—C11—H11	107.5	H17A—C17—H17B	108.0
C13—C11—H11	107.6	C17—C18—C19	111.79 (19)
C7—C11—H11	107.4	C17—C18—H18A	109.4
N2—C14—C19	108.47 (16)	C19—C18—H18A	109.5
N2—C14—C15	112.84 (16)	C17—C18—H18B	109.0
C19—C14—C15	110.73 (17)	C19—C18—H18B	109.1
N2—C14—H14	108.2	H18A—C18—H18B	107.9
C19—C14—H14	108.3	C21—C22—C23	110.91 (19)
C15—C14—H14	108.1	C21—C22—H22A	109.7
C22—C21—C20	110.87 (18)	C23—C22—H22A	109.6
C22—C21—H21A	109.4	C21—C22—H22B	109.2
C20—C21—H21A	109.6	C23—C22—H22B	109.4
C22—C21—H21B	109.4	H22A—C22—H22B	108.0
